# Footprint to prediction: low-cost, three-class foot arch screening using a Harris mat

**DOI:** 10.3389/frai.2026.1849098

**Published:** 2026-10-07

**Authors:** Hariprasad Ramachandran, Srivardan Balu, Kaushik Ramakrishna Haran, Sanjay Murugan Selvi, Sharmila Nageswaran

**Affiliations:** School of Electronics Engineering, Vellore Institute of Technology, Vellore, Tamil Nadu, India

**Keywords:** community screening, Normal Arch, Pes Cavus, Pes Planus, transfer learning

## Abstract

Arch type foot pathologies of Pes Cavus (high arch), Pes Planus (flatfoot), and Normal Arch patterns are good predictors of foot health that affect gait, posture, and predispose to musculoskeletal disorders. The research work described herein advances a computer-assisted automated classification of the foot arch from footprint images obtained on the Harris mat and transformed into contact-area visualizations through image processing, then classified using machine learning approaches, including a Convolutional Neural Network (CNN)-based model with MobileNetV2 and a Support Vector Machine (SVM) baseline for comparison. The system’s performance was validated on a dataset of 106 footprint impressions collected from 53 healthy young university students. The training set was subsequently augmented to 300 images, while evaluation was performed on 22 held-out original (non-augmented) images. The total accuracy for the CNN model was 77.3%, with an F1-score by class of 0.78 for Pes Cavus, 0.92 for Pes Planus, and 0.62 for Normal Arch. Precision values were 0.70 (Pes Cavus), 0.86 (Pes Planus), and 0.80 (Normal Arch), and recall values were 0.88, 1.00, and 0.50, respectively. The SVM model had an accuracy of 54.6%. Cohen’s Kappa of 0.658 indicated substantial agreement between model predictions and reference labels, and ROC analysis showed promising classification performance within the evaluated dataset, with AUC estimates of 0.848 for Pes Cavus, 0.990 for Pes Planus, and 0.830 for Normal Arch.

## Introduction

Foot health is a fundamental yet often underappreciated component of overall musculoskeletal wellbeing. Structural deformities of the foot arch, including Pes Planus (flatfoot) and Pes Cavus (high arch), affect a significant proportion of the population and are associated with secondary complications such as plantar fasciitis, ankle instability, lower-limb pain, gait abnormalities, and postural disorders (Carr et al., 2016). Early identification of these conditions is important for preventive healthcare, rehabilitation planning, and orthotic intervention. However, routine foot arch screening remains inaccessible in many community and primary healthcare settings due to the high cost of specialized equipment such as pedobarographs and force plates, as well as the limited availability of trained clinical personnel. Consequently, the development of low-cost, automated, and clinically accessible tools for foot arch assessment has emerged as an important objective for large-scale screening and early diagnosis, particularly in resource-constrained environments.

Foot arch morphology plays a critical role in gait biomechanics, posture, and musculoskeletal health. The three principal arch types: Pes Planus (flatfoot), Normal Arch, and Pes Cavus (high arch) are associated with distinct loading patterns and are predictors of downstream pathology including ankle instability, plantar fasciitis, and lower-limb pain (Kirby, 2017; Paecharoen and Kritsanapraneet, 2024; Coughlin and Kaz, 2009). Early and accurate classification of arch type is therefore clinically important for preventive screening, orthotic prescription (Açak, 2020; Jung et al., 2022; Menz et al., 2012), and rehabilitative planning (Chevalier and Chockalingam, 2012; Banwell et al., 2014). Despite its significance, routine foot arch assessment remains limited by reliance on costly sensor-based hardware such as pedobarographs and force plates, or on subjective visual inspection, both of which are impractical in resource-limited community health settings. Traditional footprint-based classification approaches have relied on manual assessment tools and rule-based schemes (Nikolaidou and Boudolos, 2006; Toyooka et al., 2021).

Recent advances in machine learning and computer vision have opened new avenues for automated foot morphology analysis. Convolutional neural networks (CNNs) have been successfully applied to classify foot types from plantar pressure images and from X-ray images (Chae et al., 2020; Zhao et al., 2023; Danaci et al., 2025), with related computer-vision-based classification approaches also demonstrated for foot deformity analysis (Babović et al., 2023), and related feature-extraction approaches applied to plantar pressure and gait analysis more broadly (Bai et al., 2025; Wang et al., 2019). Chemello et al. (2022) conducted a narrative review of artificial intelligence methodologies applied to screening, diagnosis, and care of the diabetic foot, highlighting the broader applicability of AI-based technologies to foot health assessment. More recently, Doğan et al. (2024) proposed a CNN–Vision Transformer (ViT) fusion model combined with ensemble learning for automatic pes planus detection from weight-bearing foot radiographs, reporting improved diagnostic performance through the integration of CNN and transformer architectures. Most recently, Vardhan et al. (2026) compared handcrafted-feature and deep learning models, including SHAP- and Grad-CAM-based explainability, for flatfoot classification from weight-bearing X-ray images. Farhan similarly applied artificial intelligence methods to foot-type classification within an IoT-enabled framework (University of Diyala and Farhan, 2025). Similar decision-tree and neural network approaches have also been applied to classify pediatric foot disorders (Choi et al., 2017). These studies demonstrate the potential of modern deep learning techniques for foot assessment; however, most rely on specialized imaging modalities such as radiographs, plantar pressure systems, or clinically controlled acquisition environments.

Despite these advances, relatively few studies using sensor-based or radiographic imaging (Chae et al., 2020; Zhao et al., 2023; Danaci et al., 2025), or AI-driven review (Chemello et al., 2022) and fusion-model (Doğan et al., 2024) approaches, have investigated automated foot arch classification using conventional Harris mat footprint impressions. The closest existing attempt, Uluöz et al. (2021), applied the Harris mat together with the Staheli and Chippaux-Smirak indices, but relied on manual index comparison rather than an automated or learned classifier. Existing approaches often require dedicated hardware, trained personnel, or imaging systems that may limit accessibility in community healthcare settings. In contrast, the proposed framework utilizes low-cost Harris mat footprints and converts them into standardized contact-area visualizations through image-processing techniques. By integrating these image-derived representations with established morphometric indices and a lightweight MobileNetV2 classifier, the proposed system provides a sensor-free, clinically interpretable, and web-deployable solution for automated foot arch assessment suitable for resource-constrained environments.

Harris mat footprints are widely used, but most existing methods rely only on grayscale intensity mapping, without standardized measurements to support it. This limits how consistent and interpretable the results are across different people. This study addresses that gap by pairing image-based contact-area visualization with established footprint indices, creating an automated, interpretable, and low-cost screening tool suitable for community health and remote monitoring settings. This study aims to: (1) develop an image-processing pipeline that converts Harris mat footprint impressions into standardized contact-area visualizations without requiring specialized pressure-sensing hardware; (2) compute established morphometric indices (the Staheli Index and Chippaux-Smirak Index) from the extracted footprint measurements, and separately develop a MobileNetV2-based transfer learning classifier operating on the contact-area visualizations, for three-class foot arch classification (Pes Planus, Normal Arch, Pes Cavus); (3) compare the CNN-based image classifier’s performance against an SVM baseline, and develop a rule-based morphometric classifier as an interpretable reference approach, for foot arch classification; (4) integrate the classification pipeline within a web-based application to demonstrate feasibility for low-cost, sensor-free foot arch screening in resource-constrained community healthcare settings. The CNN-based image classifier, leveraging learned spatial representations from the MobileNetV2 backbone, will achieve higher classification performance than the traditional machine learning (SVM) baseline.

## Methodology

### Study

A diagnostic strategy was established that merges standard foot anthropometry measured with artificial intelligence methods to categorize three types of foot arches (Pes Planus, Normal Arch, and Pes Cavus). A total of 53 healthy young adult university students participated in the study, contributing 106 static footprint impressions (both left and right feet per participant) collected using a Harris mat under standardized conditions, with informed consent obtained from all participants for academic research use. It is acknowledged that this participant pool represents a narrow demographic, and the model’s performance on older adults, pediatric populations, or individuals with pre-existing foot or gait pathologies has not been evaluated and remains a subject for future investigation. Of the 106 original footprint images, 28 belonged to the Pes Planus class, 38 to Normal Arch, and 40 to Pes Cavus, with Pes Planus representing the smallest class and Pes Cavus the largest. The images were then digitized and saved as PDFs. Data augmentation, including random rotations (±15°), horizontal flipping, and Gaussian blurring, was applied exclusively to the training set to address class imbalance, bringing each class to 100 images and resulting in a balanced dataset of 300 images total (100 per class), consistent with prior evidence that combining transfer learning with data augmentation improves prediction performance in clinical machine learning tasks more broadly (Deng et al., 2021). The Staheli Index (SI) and Chippaux-Smirak Index (CSI) were computed from the extracted morphometric dimensions using the established formulas SI = MW/HW and CSI = MW/FW × 100, following Staheli et al. (1987) and Ghazaleh et al. (2019), respectively.

Two physiotherapists with experience in foot biomechanics independently reviewed and labeled each footprint image according to standard clinical criteria, consistent with prior work assessing intra- and inter-examiner reliability of Harris mat footprint analysis (Cisneros et al., 2010). Raw agreement between the two physiotherapists was 100%. As agreement was complete, no footprints required consensus reconciliation. All data received the appropriate anonymization. Images were anonymized prior to analysis and handled in accordance with institutional ethical requirements. People with gait issues or previous injuries were not included in the study.

To prevent data leakage, dataset splitting was performed at the individual level rather than the image level. All footprint images from a given participant (both left and right feet) were assigned exclusively to one of the two subsets: training and test, ensuring that no individual contributed images to more than one subset. Left and right foot images were kept together within the same participant’s allocation. This individual-based split guarantees that the model’s performance on the test set reflects generalization to unseen individuals rather than unseen images from known participants.

### Data preprocessing and acquisition

Footprint images were obtained in PDF format through a standardized scanning procedure to maintain uniformity in all samples. The scanned footprints were then processed through a custom-built pipeline implemented in Python using the PyMuPDF and OpenCV libraries. Each image was converted to grayscale and resized to 600 × 800 pixels to ensure dimensional uniformity. Binary thresholding was performed using Otsu’s method to separate the footprint from the background. Next, the largest external contour by pixel area was extracted to identify the footprint boundary. Each Harris mat acquisition captured a single foot per page by protocol, so no image contained more than one plausible footprint-sized contour; consequently, no images required manual intervention at this step. For anatomical alignment consistency throughout samples, Principal Component Analysis (PCA) was used to orient the foot so that the principal axis was in the vertical direction (Lucas et al., 2018). This normalization allowed for accurate spatial comparison and region-specific analysis throughout the dataset.

While CNNs are capable of learning directly from raw images, the Otsu thresholding and contour-based segmentation steps serve to standardize the input space by isolating the footprint region from variable background noise introduced during scanning. The preprocessing was adopted based on its established role in removing scanner-specific artefacts and improving input standardization in prior footprint analysis literature. The segmented and PCA-aligned footprint images are then used as input to MobileNetV2.

### Foot region segmentation and index calculation

Each aligned foot silhouette image was divided into three anatomical regions: the posterior 30% (heel), mid 30% (midfoot), and anterior 40% (forefoot) of the bounding box. These areas were marked proportionally to the vertical size of the footprint. For visualizing contact-area distribution, binary images were smoothed using a Gaussian function and projected onto heatmaps using the ‘jet’ colormap of Matplotlib (Voronov et al., 2009). The color intensities of the heatmap reflect relative pixel density derived from the Harris mat footprint image and serve as a surrogate indicator of contact-area concentration, not as a direct measure of biomechanical pressure magnitude. True plantar pressure measurement requires calibrated force-sensing hardware such as pedobarographs or pressure platforms. This image-based visualization approach is consistent with prior work using digitized Harris mat recordings as qualitative foot loading indicators (Voronov et al., 2009). Although the proposed contact-area visualization does not represent calibrated plantar pressure measurements, its qualitative patterns are consistent with established biomechanical understanding of foot arch behavior. Flat feet (Pes Planus) are characterized by increased midfoot contact, while high arches (Pes Cavus) exhibit reduced midfoot contact and increased forefoot and heel loading. The generated contact-area visualizations in this study demonstrated these expected distribution patterns across classes, providing indirect validation of the proposed approach. This observation aligns with prior studies that utilize footprint-based intensity distributions as qualitative indicators of plantar loading patterns (see Figures 1–3).

**Figure 1:**
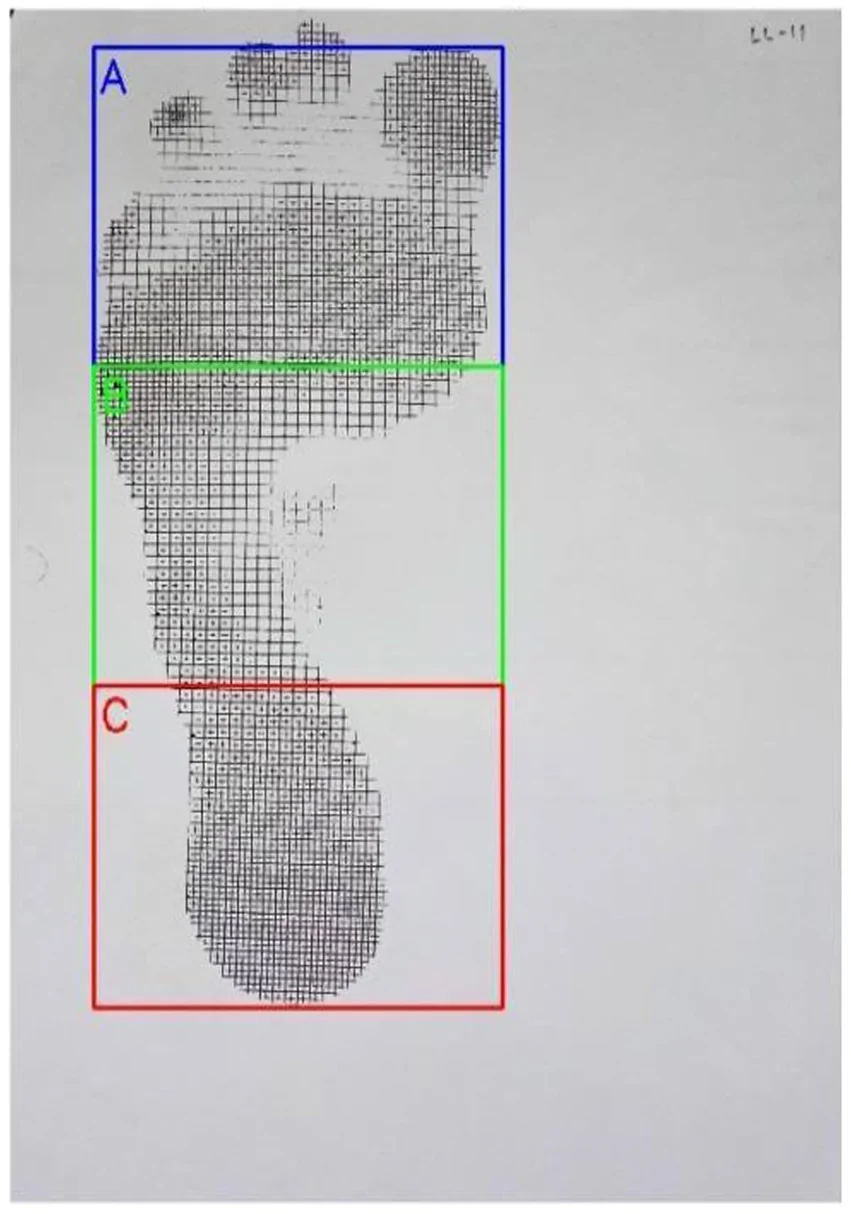
Measurements taken in Areas A, B, and C of the foot, corresponding to the forefoot, midfoot, and hindfoot regions, are used to derive the Staheli Index and Chippaux-Smirak Index.

**Figure 2:**
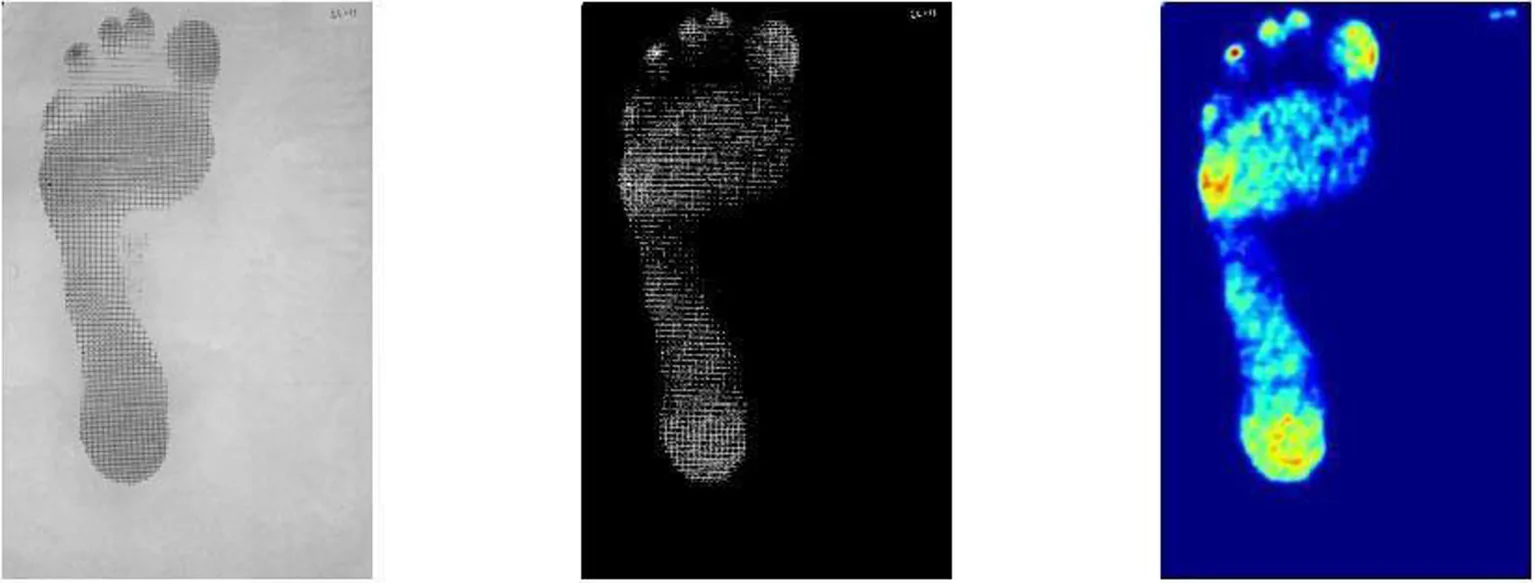
Arch classification analysis utilizes contact-area visualization, shown through the combination of original Harris mat footprint images (left), grayscale processing (center), and contact-area heatmap (right).

**Figure 3:**
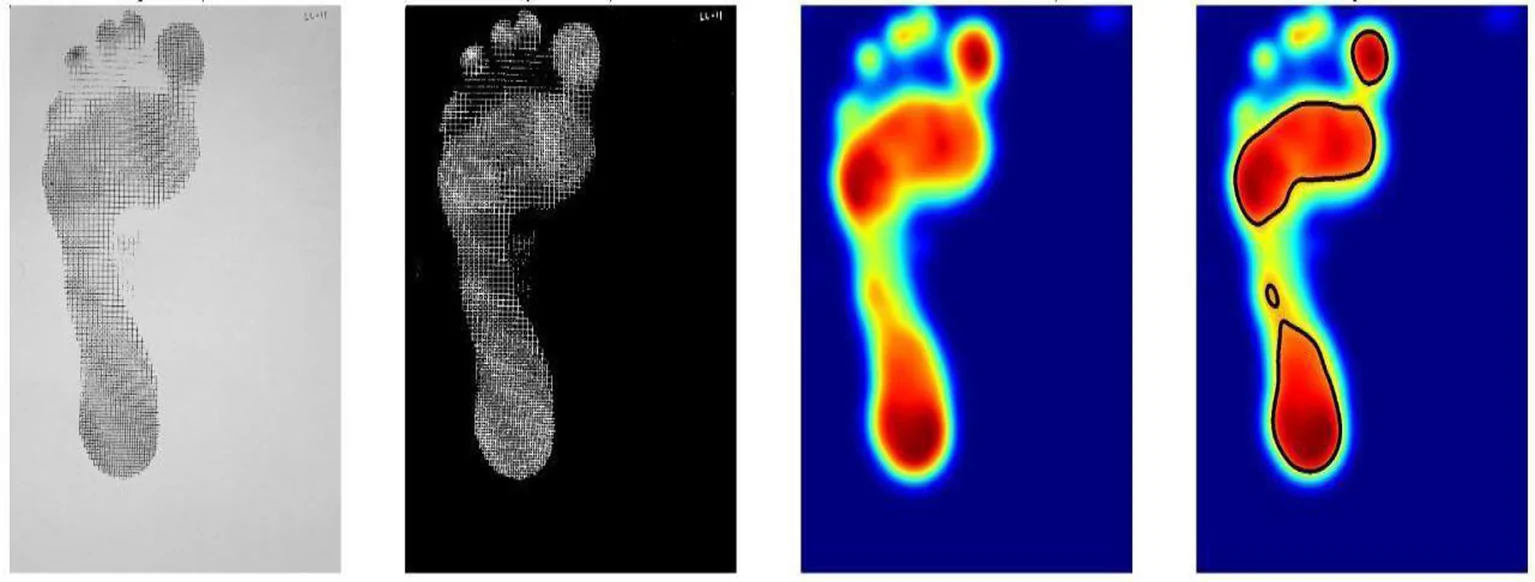
Sequential visualization of footprint analysis showing how the image evolves from a grayscale footprint to a thresholded binary mask, leading to a Gaussian-smoothed contact-area heatmap followed by high-density contours placed on the contact-area visualizations.

Quantitative morphometric measurements were computed for classification:

- Midfoot Width (MW): Narrowest width of the midfoot area
- Heel Width (HW): Widest width in the heel area
- Forefoot Width (FW): Maximum width in the forefoot area

Each pixel measurement was normalized to inches from DPI scaling.

Two classic clinical indices were subsequently calculated:

- Staheli Index (SI):

SI=Midfoot Width/Heel Width

- Chippaux-Smirak Index (CSI):

CSI=(Midfoot Width/Forefoot Width)×100.

### Hybrid rule-based classification

The hybrid rule-based classifier applied anatomical measures as well as regional intensity features derived from heatmaps. Mean pixel intensity of the center 30% horizontal band (arch region) was computed after smoothing and normalization.

Midfoot width thresholds were derived empirically from the observed distribution of the dataset. Pes Planus footprints had a mean midfoot width of 4.43 inches (SD = 0.67 in, *n* = 28), compared to 2.21 inches (SD = 0.57 in, *n* = 38) for Normal Arch and 2.16 inches (SD = 0.47 in, *n* = 40) for Pes Cavus. Midfoot width cleanly separated Pes Planus from the pooled Normal Arch and Pes Cavus group, with a threshold of 3.45 inches achieving 98.1% classification accuracy for this split. However, midfoot width alone did not distinguish Normal Arch from Pes Cavus, as these two classes showed near-identical width distributions; this distinction instead relied on arch-region intensity, as described above.

- Pes Planus (Flat Foot): Midfoot Width > 3.45 inches, together with increased arch-region intensity.
- Pes Cavus (High Arch): Midfoot Width ≤ 3.45 inches, together with low arch-region intensity.
- Normal Arch: Midfoot Width ≤ 3.45 inches, without the low arch-region intensity pattern associated with Pes Cavus (Ghazaleh et al., 2019).

It should be noted that fixed inch-based thresholds may not generalize across populations with varying anthropometric characteristics such as age, sex, body size, and ethnicity. These thresholds were therefore used as approximate decision boundaries within the context of the present dataset and should be interpreted as dataset-specific approximations rather than universally applicable clinical cutoffs.

The above method effectively integrated morphometric geometry with image-derived contact-area intensity values for classification (Gwani et al., 2017; Kirby, 2017; Voronov et al., 2009). While the heatmap component provides relative contact-density information rather than calibrated pressure data, it contributes to a useful additional feature dimension alongside the morphometric indices.

### Automated foot arch classification framework

To enable automated classification of foot arch types, a deep learning-based approach was implemented using a transfer learning framework with MobileNetV2 as the backbone architecture, consistent with established CNN architectures used for medical image classification tasks (Alzubaidi et al., 2021). MobileNetV2 was selected due to its lightweight design, computational efficiency, and suitability for deployment in resource-constrained environments. Compared to deeper architectures, it achieves competitive performance with significantly fewer parameters, making it well-suited for small-to-medium-sized medical imaging datasets. Prior studies have demonstrated the effectiveness of transfer learning-based models for foot deformity classification tasks (Doğan et al., 2024).

The MobileNetV2 model receives the preprocessed footprint image (224 × 224 pixels) as its sole input and learns spatial feature representations through its convolutional layers. Morphometric features (MW, HW, FW, SI, CSI) are not fed into the CNN at any stage; they are used solely by the separate rule-based classifier described above. The CNN-based image classifier, the rule-based morphometric classifier, and the SVM baseline are evaluated and compared independently in the Results section. As all inputs across these three classifiers are derived from a single acquisition modality, the scanned Harris mat footprint, the overall framework is referred to as a combined morphometric and image-based framework.

Following a prior round of review, the dataset was divided into training and testing subsets using an 80:20 split, with stratified sampling to preserve class distribution and a fixed random seed (42) to ensure reproducibility. No separate validation subset was carved out. As a result, early stopping (monitoring test-set loss, with a patience of 8 epochs and restoration of the best-performing weights), model checkpointing, and learning-rate reduction were monitored against test-set performance at each epoch, rather than against an independent validation set. Because test-set performance therefore informed these training decisions, the reported test accuracy should be interpreted as a single held-out estimate obtained under this constraint, rather than a fully blind evaluation; this is discussed further as a limitation.

Given the small test set (22 images), the accuracy estimate has limited statistical precision: the exact binomial 95% confidence interval spans approximately 37 percentage points. The study should therefore be regarded as exploratory and hypothesis-generating, with confirmatory evaluation on a larger dataset planned for future work.

The model was trained using the Adam optimizer with a learning rate of 0.001 and categorical cross-entropy as the loss function. A batch size of 11 and a maximum of 20 epochs were used. A dropout layer with a rate of 0.3 was incorporated in the fully connected layers to improve generalization. The MobileNetV2 backbone was initialized with pretrained ImageNet weights, with the convolutional base partially frozen during initial training. The final classification head consisted of a global average pooling layer followed by a fully connected dense layer with dropout (0.3) and a softmax output layer for multi-class classification. Input images were resized to 224 × 224 pixels before training. Data augmentation techniques, including random rotation (±15°), width and height shifts (±10%), zoom (±10%), and horizontal flipping, were applied exclusively to the training set to enhance model generalization. For comparative evaluation, a Support Vector Machine (SVM) classifier was implemented using handcrafted features derived from the footprint images (including geometric descriptors, Hu moments, and texture features). Hyperparameters were selected via grid search with 5-fold stratified cross-validation on the training set only, yielding a sigmoid kernel with C = 1.0, gamma = “scale,” and no class weighting. The SVM was evaluated on the same 22-image held-out test set used for the CNN and rule-based classifiers. Traditional machine learning approaches, such as SVM, rely on manually engineered features and may not capture complex spatial patterns as effectively as deep learning models.

### System integration and web deployment

To provide an overview of the proposed framework, Figure 4 illustrates the software architecture of the foot arch assessment system, while Figure 5 presents the scientific workflow of the automated footprint analysis pipeline. Together, these diagrams summarize the interaction between the web-based application, machine learning components, and the sequence of processing steps used for foot arch classification.

**Figure 4:**
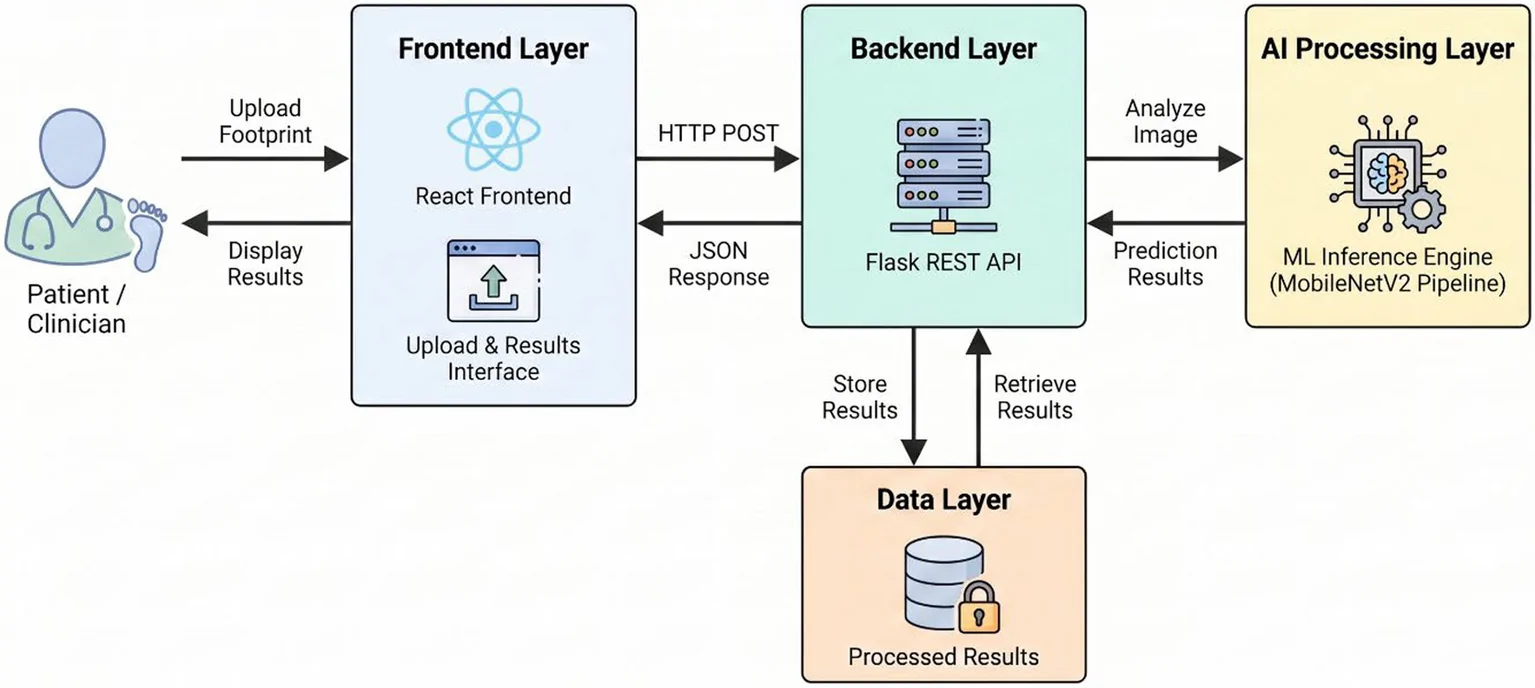
Software architecture of the foot arch assessment system. Created in BioRender. R, H. (2026) https://BioRender.com/nn8e5y8.

**Figure 5:**
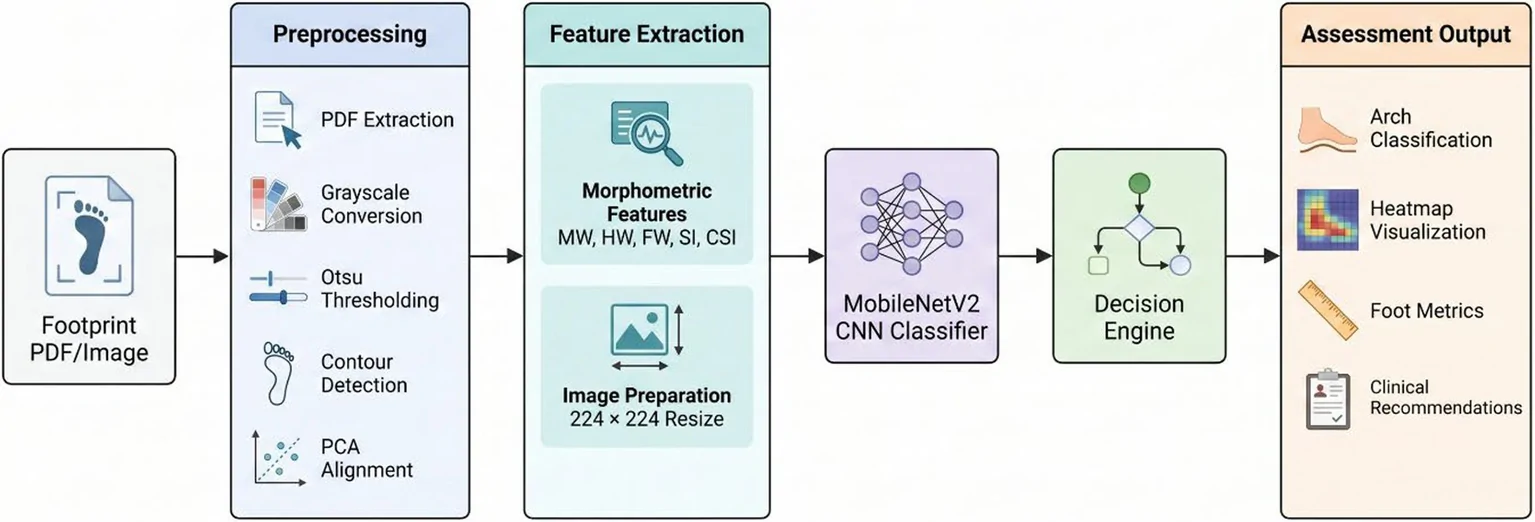
Scientific workflow for automated foot arch assessment. Created in BioRender. R, H. (2026) https://BioRender.com/h16mbae.

The software architecture consists of a React-based frontend, a Flask REST API backend, a MobileNetV2-based inference engine, and a data storage layer. The frontend enables footprint upload and result visualization, while the backend manages preprocessing, model inference, and communication between system components.

The scientific workflow begins with footprint acquisition and preprocessing, including grayscale conversion, thresholding, contour extraction, and PCA-based alignment. Morphometric features and image representations are subsequently extracted and analyzed using the MobileNetV2 classifier, followed by decision generation and presentation of diagnostic outputs through the web application.

Further a full-stack web application was integrated with the classification pipeline. A React frontend provided a responsive user experience for patients and physicians, while a Flask backend handled image ingestion, preprocessing, and model inference. Role authentication, raw and processed image thumbnails, annotated heatmap visualization, and longitudinal tracking for recurring users were among the features. Along with the indexes under “Foot Metrics” and lifestyle suggestions under “Recommendations” based on the kind of foot, the web application displays the patient’s foot breadth and length (Figure 6). It does so with a code integrated into the web application that calculates the foot length and width using dpi in accordance with the pixel size of the footprint found using OpenCV (cv2.minAreaRect() - a rotation-aware bounding box). The web application described here is a research prototype developed to demonstrate technical feasibility, rather than a clinical-grade deployment; formal data storage, retention, and access-governance arrangements would need to be established prior to any real-world deployment (Barbaria et al., 2025).

**Figure 6:**
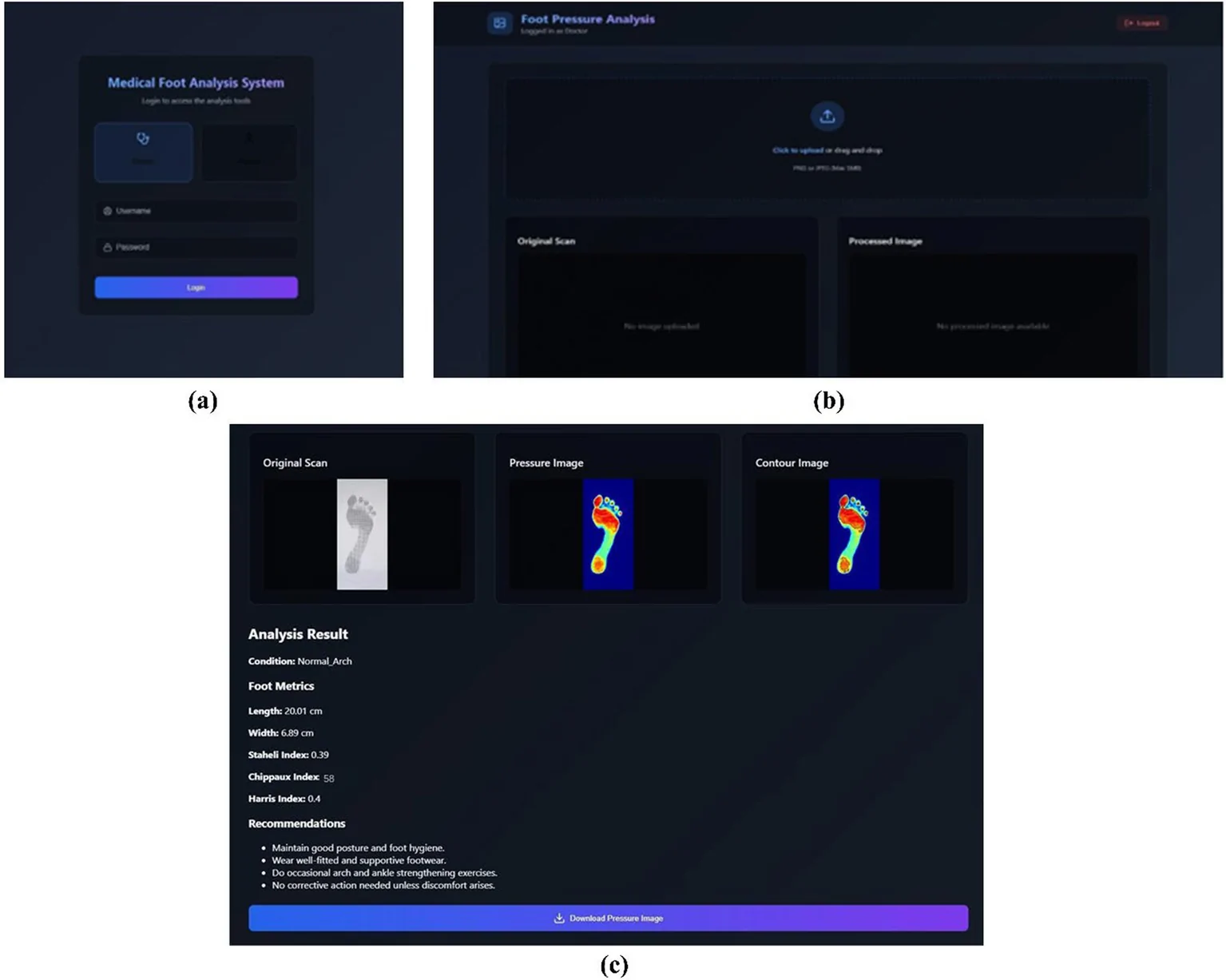
Web-based foot arch assessment system. The user workflow of the proposed application showing **(a)** role-based authentication, **(b)** footprint upload and automated processing, and **(c)** classification results with contact-area visualizations, foot metrics, confidence scores, and personalized recommendations.

The largest contour of the input is identified as a footprint, and the longer side of the rotated rectangle is classified as foot length, and the shorter side as foot width. These values are converted from pixels to cm using the dpi of the image (see Figure 7; Tables 1, 2).

**Figure 7:**
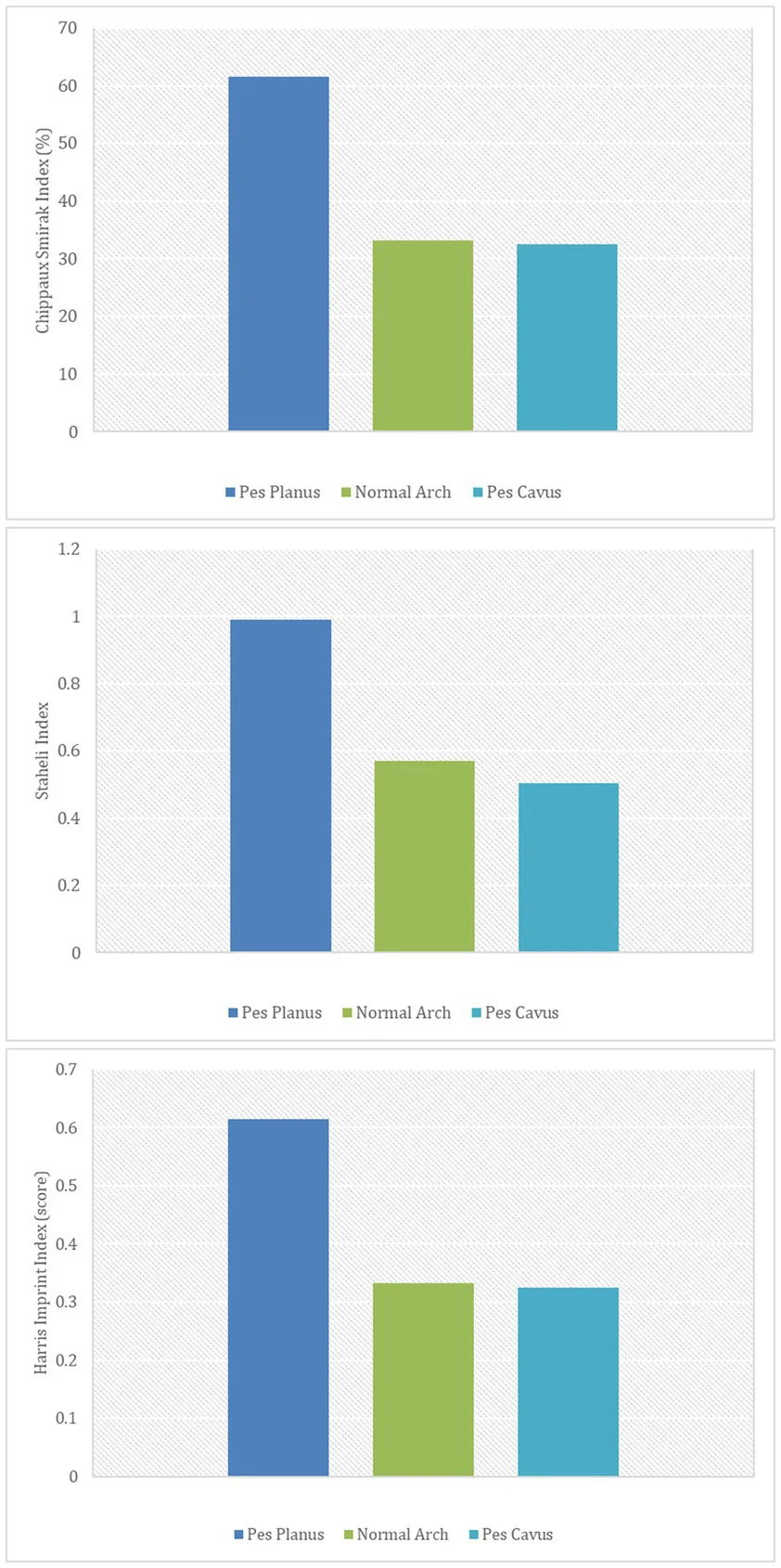
Foot indices across Pes planus, Normal arch, and Pes cavus.

**Table 1:** Summary statistics for foot arch indices.

Index	Mean	Std dev	Median
Chippaux-Smirak index	40.44	14.33	35.91
Staheli index	0.656	0.225	0.591
Harris index	0.404	0.143	0.359

**Table 2:** Summary statistics for foot measurements.

Measurement	Mean	Std dev	Median
Foot length (cm)	23.16	1.15	22.84
Midfoot width (cm)	7.06	2.90	5.95

### Statistical evaluation

Multi-class confusion matrix analysis was used to generate a set of established statistical metrics that were used for statistical analysis. Cohen’s kappa coefficient, F1-score, accuracy, sensitivity (recall), specificity, precision, and the area under the Receiver Operating Characteristic curve (ROC-AUC) were among them. All evaluations were performed on a held-out test set of 22 images drawn exclusively from original (non-augmented) footprints, representing 11 participants (4 Normal Arch, 4 Pes Cavus, and 3 Pes Planus), each contributing two footprint images (left and right foot), across three arch classes. The accuracy was measured by the number of correctly classified samples compared to all the predictions. Sensitivity (recall) was the system’s ability to detect all relevant examples for each type of foot arch, while specificity was the system’s ability to reject non-relevant samples. Accuracy was employed to determine the ratio of correct identifications to the total predictive positives. ROC curves were constructed using a one-vs-rest (OvR) strategy, wherein each class was treated as the positive class and the remaining two classes as negative. The predicted class probabilities from the softmax output layer were used as the decision score for each threshold point. AUC was computed using the trapezoidal rule via scikit-learn’s roc_auc_score function with the multi_class = ‘ovr’ parameter. The F1-score, which is the harmonic mean of precision and recall, played a specific role in this setting because of the possibility of class imbalance, where false positives and false negatives cannot be discarded. Cohen’s kappas were computed to measure the agreement between predicted and actual classifications over that expected by chance, providing a robust inter-rater reliability measure in the multi-class case. Furthermore, ROC curves based on a one-vs-rest strategy were constructed for each class to plot sensitivity versus 1-specificity for different thresholds and calculate ROC-AUC per model to measure the discriminating power of the same.

## Results and discussion

The proposed framework integrates morphometric features with image-based contact-area visualization to classify foot arch types, including Pes Planus, Pes Cavus, and Normal Arch.

For the CNN model, the accuracy was 77.3%, with a Cohen’s Kappa of 0.658, indicating substantial agreement between the model’s predictions and the reference labels. The class-wise F1 scores based on the confusion matrix were 0.78 for Pes Cavus, 0.92 for Pes Planus, and 0.62 for Normal Arch. The test accuracy of the SVM model was 54.6% (see Figures 8–11).

**Figure 8:**
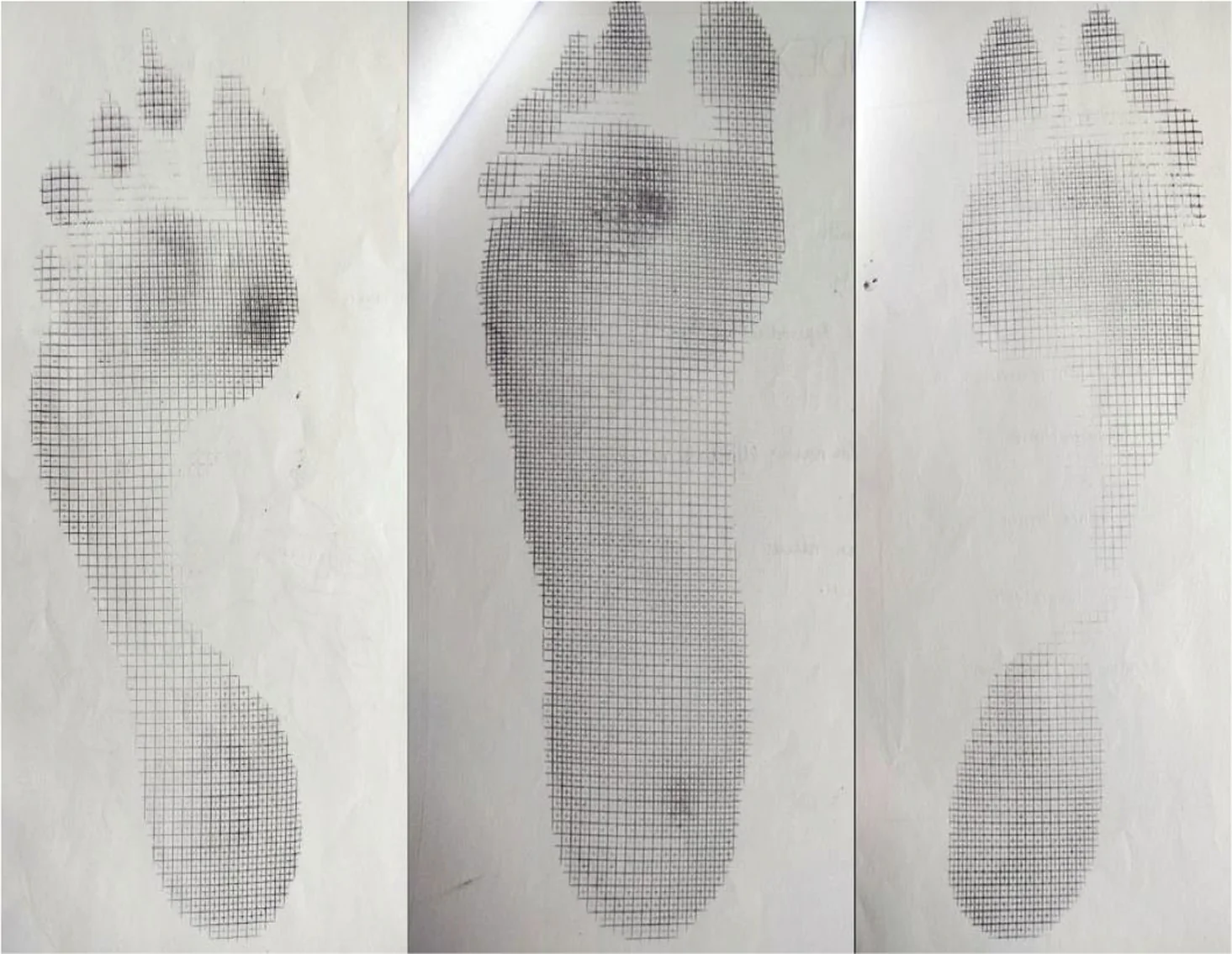
A visual representation showing Pes Planus in the middle, while Normal Arch occupies the left section, and Pes Cavus stands on the right to show structural variations between these classes.

**Figure 9:**
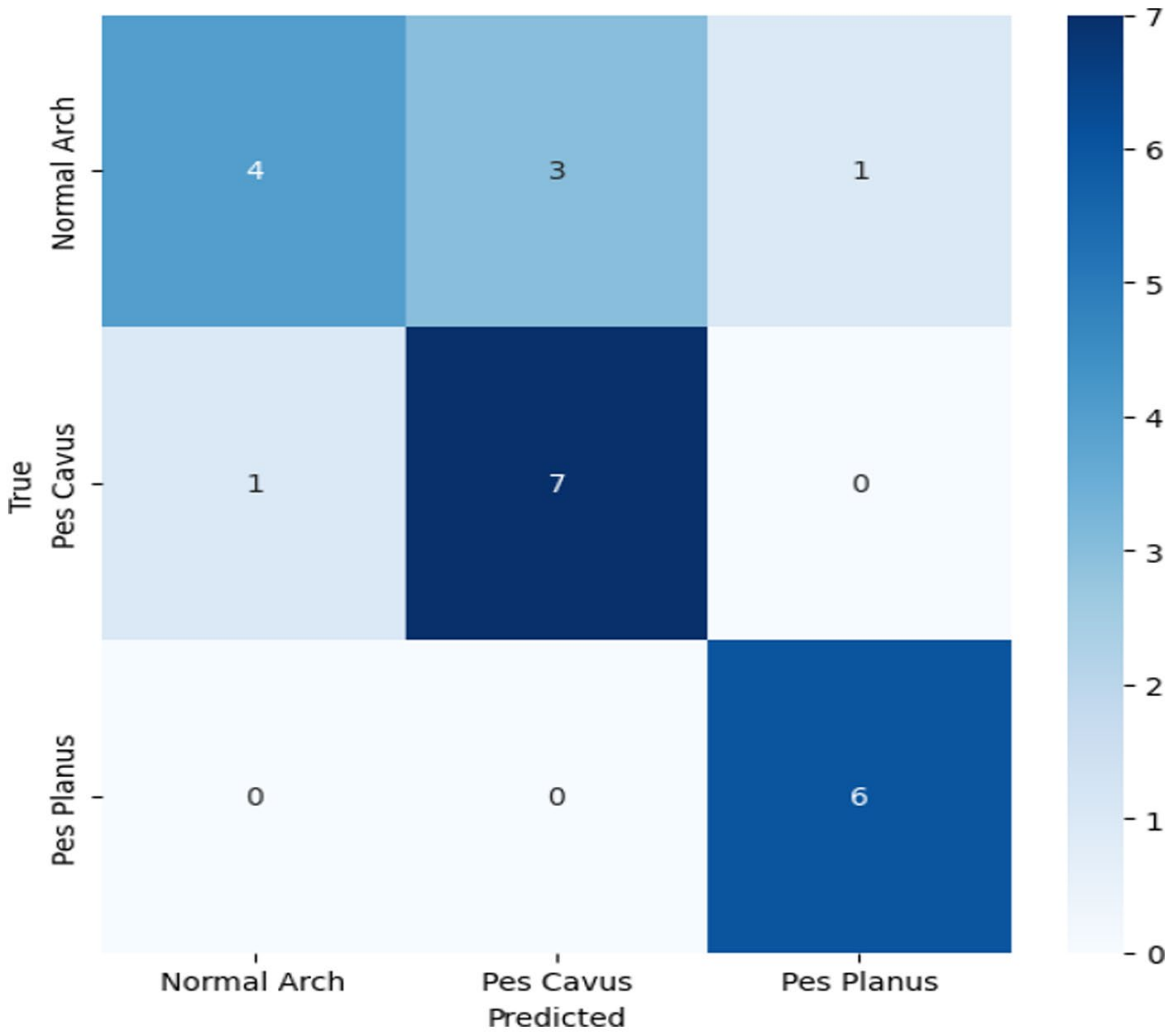
Confusion matrix illustrating the classification performance of the CNN model across the three-foot arch classes. Row totals (true class n): Normal Arch = 8, Pes Cavus = 8, Pes Planus = 6. Column totals (predicted class n): Normal Arch = 5, Pes Cavus = 10, Pes Planus = 7. Total N = 22.

**Figure 10:**
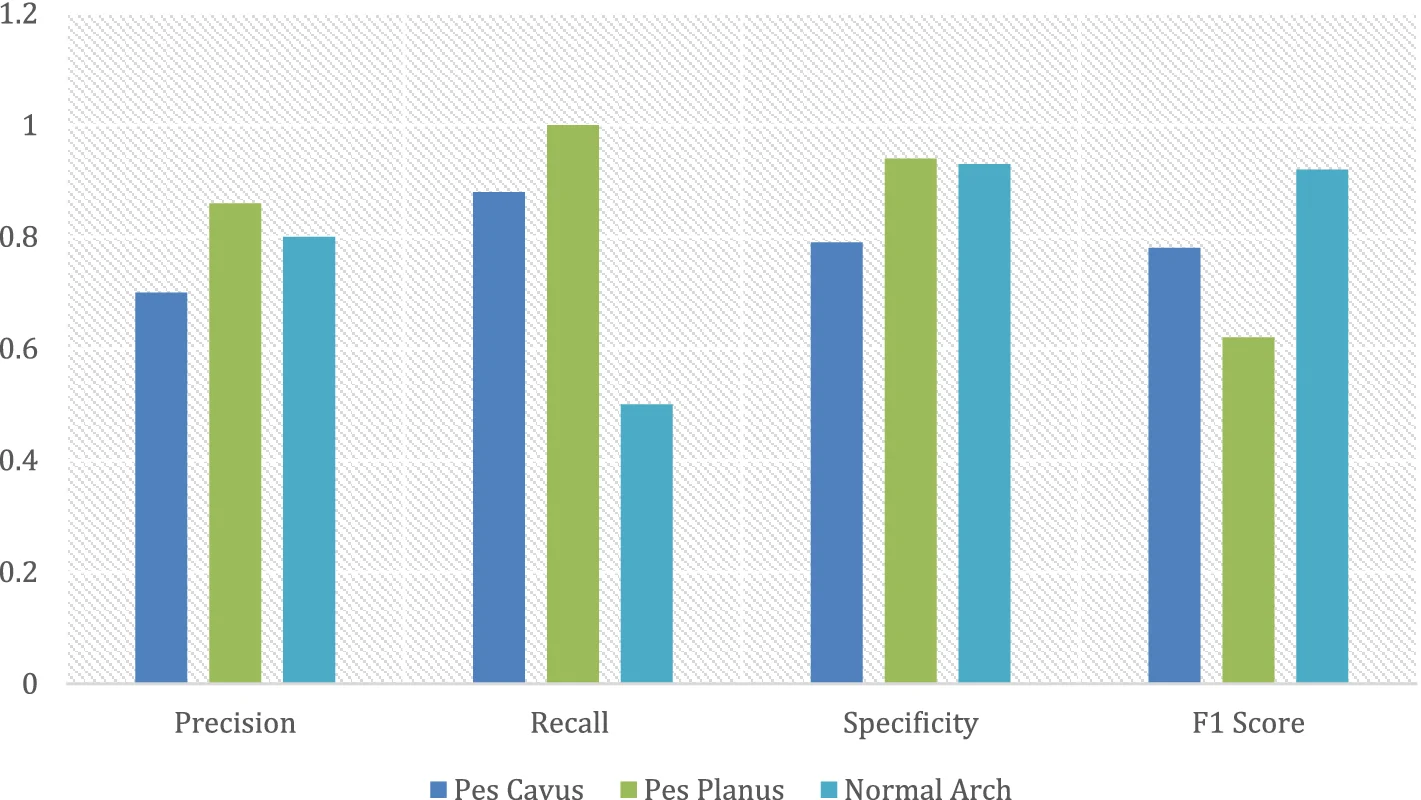
Precision, Recall, Specificity, and F1 scores comparison graphically.

**Figure 11:**
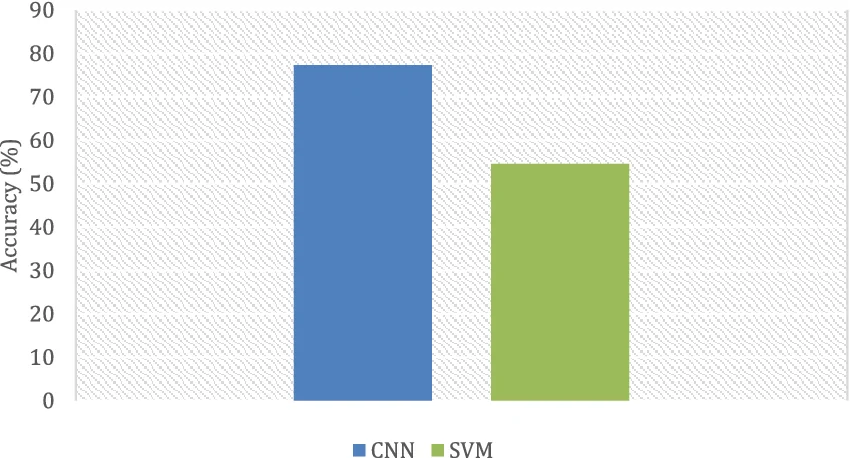
Accuracy comparison between the CNN and SVM models.

Table 3 shows how the model performed for each category. The model demonstrated competitive performance across evaluation metrics, with Pes Planus achieving the highest F1 score. Despite slightly lower performance for the Normal Arch class, the model was able to capture subtle distinctions in footprint morphology. Figure 12 demonstrates the model’s discriminative ability across the three foot-arch classes, with AUC values indicating strong class separation performance.

**Table 3:** Precision, recall, specificity, and F1 scores by class, with sample size.

Metric	Pes Cavus	Pes Planus	Normal Arch
Precision	0.7	0.86	0.8
Recall	0.88	1	0.5
Specificity	0.79	0.94	0.93
F1 Score	0.78	0.92	0.62
*n* (test set)	8	6	8

Overall accuracy: 77.3% (17/22).

**Figure 12:**
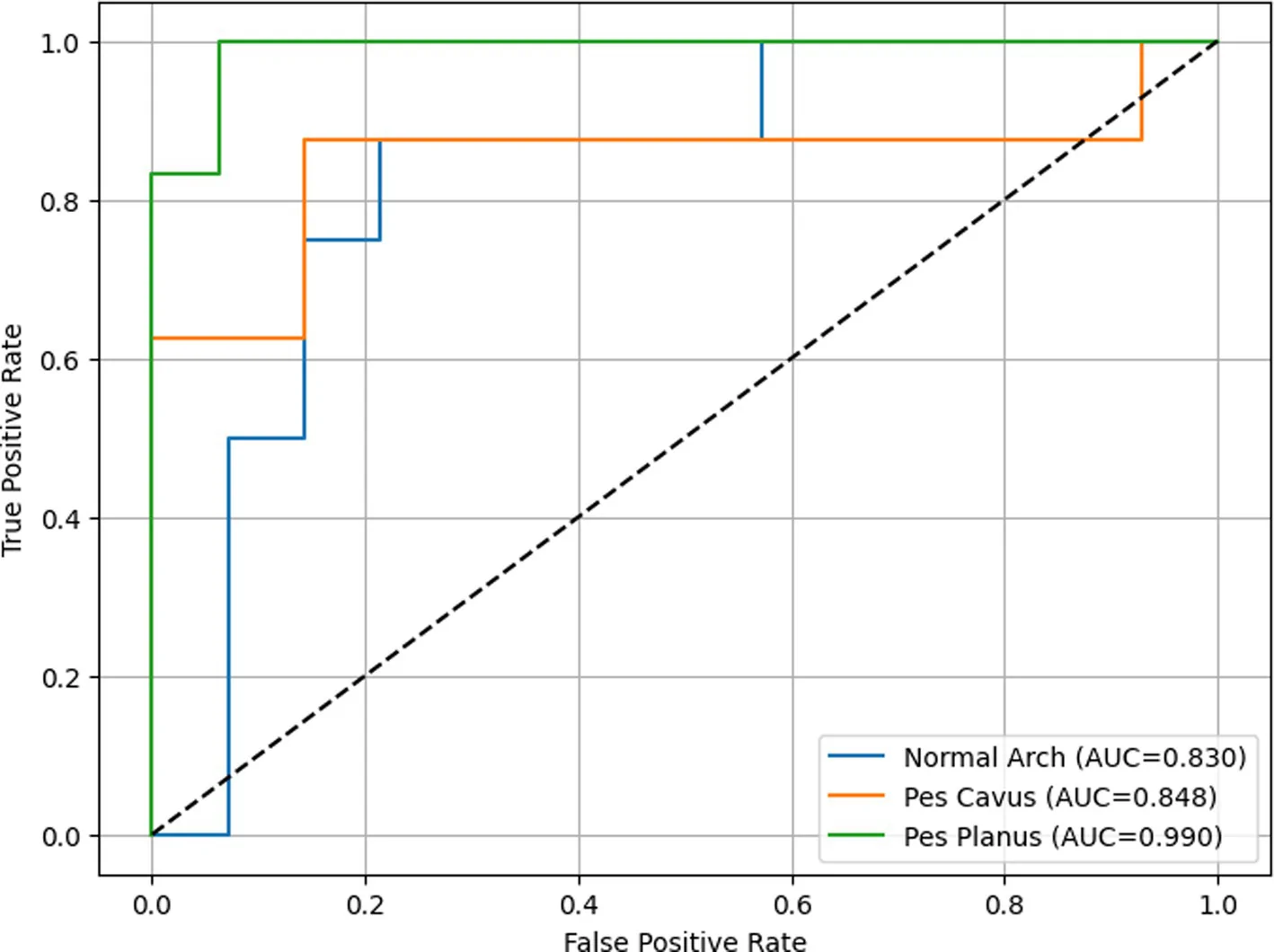
ROC curves plotted for each foot type evaluation group show how the model separates classes with effective true positive classification abilities. These curves are computed using a one-vs-rest (OvR) strategy with softmax class probabilities as decision scores.

Given a test set of 22 images, the observed accuracy of 77.3% (17/22 correct) corresponds to an exact binomial 95% confidence interval of [54.6, 92.2%], a range of 37.5 percentage points. This wide interval reflects the limited size of the held-out test set and indicates that while the point estimate is promising, the precision of this estimate is limited; a substantially larger test set would be required to narrow this range. Confidence intervals for class-level F1 scores were not computed given the small per-class sample counts; these should be interpreted with appropriate caution.

The model performance was evaluated using a held-out test set derived from an 80:20 stratified train– test split, ensuring that class distribution was preserved across both subsets. As noted in the Methods, no independent validation subset was available; test-set performance was therefore used to guide early stopping, checkpoint selection, and learning-rate scheduling during training. The reported accuracy should accordingly be interpreted as a single held-out estimate obtained under this constraint, rather than a fully blind, unbiased evaluation.

Additionally, Receiver Operating Characteristic (ROC) curves for all classes through a one-vs-rest approach were calculated. Area Under the Curve (AUC) values were 0.848 for Pes Cavus, 0.990 for Pes Planus, and 0.830 for Normal Arch (see Table 4).

- The macro-average AUC was provided as:

AUC_macro=(0.848+0.990+0.830)/3=0.889

**Table 4:** Receiver operating characteristic (ROC) and area under curve (AUC) analysis for foot arch classification.

Class	TPR	FPR	AUC
Pes Cavus	0.875	0.214	0.848
Pes Planus	1	0.063	0.990
Normal Arch	0.5	0.071	0.830

The rule-based hybrid classifier based on rules, which integrates morphometric characteristics with contact-area intensity values derived from contact-area visualization, provides an interpretable baseline for classification.

As noted above, the observed accuracy of 77.3% corresponds to an exact binomial 95% confidence interval of [54.6, 92.2%]. The lower bound of this interval approaches a level that would not support clinical use, underscoring the exploratory, hypothesis-generating nature of the present findings.

These results indicate that the system is effective in distinguishing between the three classes, particularly for Pes Planus and Pes Cavus, while relatively lower sensitivity is observed for Normal Arch. Pes Planus was classified with comparatively higher performance (F1 = 0.92), likely due to more distinct morphological characteristics. In contrast, Pes Cavus had somewhat lower performance (F1 = 0.78) than Pes Planus, likely due to the likelihood of morphological resemblance with the Normal Arch class in some cases.

This approach, although simple, proved effective in identifying clinically meaningful differences in footprint shape and contact-area distribution patterns. It should be noted that the heatmap-derived intensity values reflect image-based contact density rather than sensor-measured pressure, consistent with prior studies using digitized Harris mat recordings as qualitative foot loading indicators (Voronov et al., 2009). The accessibility of the deployed web application highlights the feasibility of translating the proposed approach into an accessible tool. However, further validation is required before practical deployment in clinical settings.

The application currently returns a classification, foot metrics, a confidence score, and personalized recommendations to the user, so it is important to state plainly what stage of development this represents and what would need to happen both before and after any real deployment. Using a staged AI development-validation-implementation framework (Dergaa et al., 2026), the tool has reached only the technical feasibility stage: a functioning pipeline evaluated on a single-institution dataset of 22 held-out images, without external validation, prospective evaluation, or calibration of its confidence scores. Before any recommendation is shown to a patient, external validation, calibration analysis, a prospective comparison against clinician judgment, and sign-off from a clinical governance body would be the minimum requirements. After deployment, drift as the tool meets new scanners, operators, and populations would also need to be actively managed. Adapting the Learn-Predict-Monitor-Detect-Correct framework for clinical AI (Boussi Rahmouni et al., 2025), this would require logging every prediction and, where available, its true outcome; periodic review for confidence drift or shifts in the predicted class distribution; and a defined retraining trigger with a named reviewer responsible for approving any updated model. None of these pre- or post-deployment mechanisms are currently implemented; the tool should accordingly be regarded, at its current stage, as a research prototype rather than a clinical decision-support system.

This study has several limitations. First, the morphometric thresholds used (e.g., midfoot width cutoffs) were derived empirically from this dataset and may not generalize across populations differing in age, sex, body size, or ethnicity. A concrete alternative is to include a body-size or developmental measure, such as age or foot length, as an input to a learned classifier, so the decision boundary can adjust to that measure instead of using one fixed cutoff for everyone. Ghouili et al. used this approach successfully, along with methods like cost-sensitive learning or synthetic oversampling for class imbalance, in a similar pediatric screening problem (Ghouili et al., 2026). Second, the dataset (106 images, 53 participants) is relatively small and demographically homogeneous, comprising healthy young adult university students, which limits both the statistical reliability of the reported metrics and generalizability to older adults, children, or individuals with gait pathologies. Third, the intensity-based visualization reflects image contact density rather than calibrated plantar pressure, limiting direct biomechanical interpretation. Fourth, early stopping and checkpoint selection were guided by test-set performance rather than a separate validation set, so the reported accuracy is likely somewhat optimistic relative to a fully blind evaluation. Fifth, with only 22 test images, the accuracy estimate is imprecise rather than biased in a particular direction: the exact binomial 95% confidence interval spans about 37 percentage points, so the true accuracy could plausibly sit anywhere within that range. Sixth, the model’s confidence scores have not been calibrated, which does not affect the accuracy figure itself but means the confidence values shown to users should not yet be read as calibrated probabilities. Finally, all data came from a single standardized acquisition protocol. Validation on independently collected data is a priority for future work, alongside larger datasets and integration with sensor-based pressure measurements, building on the substantial existing body of work in wearable and motion-analysis pipelines (Souaifi et al., 2025).

Table 5 summarizes these results alongside three comparable prior studies. The present study addresses a more challenging three-class problem on a smaller dataset than the binary-classification studies of Zhao et al. (2023) and Doğan et al. (2024), while using a lower-cost, sensor-free acquisition method than the pressure-sensor and radiographic modalities used in the comparison studies.

**Table 5:** Comparison with prior foot-type classification studies.

Study	Sample size	Imaging modality	Classes	Accuracy
Chae et al. (2020)	96 subjects	Plantar pressure system	3	92.6%
Zhao et al. (2023)	125 subjects	Plantar pressure system	2	82.6%
Doğan et al. (2024)	421 subjects	X-ray	2	96.8%
Present study	53 subjects	Harris mat	3	77.3%

A conceptual comparison can be drawn between the proposed framework and recent deep learning-based approaches for diabetic foot assessment, such as image-based diabetic foot ulcer (DFU) risk models developed on publicly available datasets (Yap et al., 2021; Yap et al., 2023). These models primarily rely on visual features extracted from foot images to classify ulcer presence or severity using convolutional neural networks. In contrast, the proposed approach integrates morphometric measurements with image-derived contact-area visualization, enabling the capture of both geometric and spatial characteristics of the foot. While DFU models focus on pathological lesion detection, the present work addresses structural foot morphology classification. This distinction highlights the complementary nature of the proposed framework, which may serve as an early-stage screening tool prior to the onset of more severe conditions such as ulcers.

## Conclusion

This study introduces a framework that pairs morphometric feature extraction with image-based contact-area visualization and machine learning classification to provide a screening-level classification of foot arch type from single footprint images. Using rule-based clinical knowledge alongside modern machine learning approaches provides both interpretability and classification performance, bridging traditional clinical assessment with data-driven classification. The CNN model achieved 77.3% classification accuracy across all three target classes within the evaluated dataset and outperformed the SVM baseline (54.6%); generalizability to broader populations and clinical settings requires further validation on larger, more diverse cohorts. Its integration into a web-based platform demonstrates the technical feasibility of the approach; however, clinical deployment will require prospective validation on diverse and clinically representative populations. In its current form, a classifier of this accuracy is best suited to a low-resource community screening setting where no podiatrist or radiographic imaging is readily available: a community health worker could use a Pes Planus or Pes Cavus classification to prioritize an individual for referral to a specialist for confirmatory assessment, while treating a Normal Arch classification as a lower-priority signal rather than a definitive rule-out, given its recall of 0.50. Future work will target improved diagnostic resolution using dynamic gait analysis, increased and more diverse datasets, and the addition of sensor-based biomechanical measurements, building on the substantial body of existing work in wearable and motion-analysis pipelines. These additions are expected to strengthen the clinical utility of the system as a tool in preventive and rehabilitative podiatric practice. Also, expansion of the dataset to include clinically diverse populations, including older adults, pediatric patients, and individuals with gait pathologies, remains a priority for future work.

## Data Availability

The original contributions presented in the study are included in the article/supplementary material, further inquiries can be directed to the corresponding author.
